# Anti-proliferative effects of *Bifidobacterium adolescentis *SPM0212 extract on human colon cancer cell lines

**DOI:** 10.1186/1471-2407-8-310

**Published:** 2008-10-27

**Authors:** Do Kyung Lee, Seok Jang, Mi Jin Kim, Jung Hyun Kim, Myung Jun Chung, Kyung Jae Kim, Nam Joo Ha

**Affiliations:** 1 Department of Pharmacy, Sahmyook University, Seoul 139-742, Republic of Korea; 2 Department of Life Science, Sahmyook University, Seoul 139-742, Republic of Korea; 3 Cellbiotech, Co. Ltd., Seoul 157-030, Republic of Korea

## Abstract

**Background:**

Lactic acid bacteria (LAB) are beneficial probiotic organisms that contribute to improved nutrition, microbial balance, and immuno-enhancement of the intestinal tract, as well as anti-tumor activity. The aim of the present work was to study the growth inhibition of tumor cells by butanol extract of *Bifidobacterium adolescentis *isolated from healthy young Koreans.

**Methods:**

The anti-proliferative activity of *B. adolescentis *isolates was assessed by XTT assays on three human colon cancer cell lines (Caco-2, HT-29, and SW480). The effects of *B. adolescentis *SPM0212 butanol extract on tumor necrosis factor-α (TNF-α) and nitric oxide (NO) production were tested using the murine macrophage RAW 264.7 cell line.

**Results:**

The butanol extract of *B. adolescentis *SPM0212 dose-dependently inhibited the growth of Caco-2, HT-29, and SW480 cells by 70%, 30%, and 40%, respectively, at 200 μg/mL. Additionally, the butanol extract of *B. adolescentis *SPM0212 induced macrophage activation and significantly increased the production of TNF-α and NO, which regulate immune modulation and are cytotoxic to tumor cells.

**Conclusion:**

The butanol extract of *B. adolescentis *SPM0212 increased activity of the host immune system and may improve human health by helping to prevent colon cancer as a biological response modifier.

## Background

Colon cancer is a serious health problem and remains the leading cause of cancer mortality throughout the world [1]. Colon cancer incidence has rapidly increased as dietary patterns have changed to contain high fat, high protein, low carbohydrate, and low fiber [2,3]. Colon cancer is the second most common cancer in Korea [4]. Despite recent advances in our understanding of the biological processes resulting in the development of cancer, there remains a need for new and effective agents to control this disease.

Microorganisms, such as *Mycobacterium bovis*, *Streptococcus pyogenes*, *Corynebacterium parvum*, and cellular components of these bacteria have been used as biological response modifiers (BRM) and are beneficial adjuvants to cancer chemotherapy, increasing remission rates and disease-free intervals. However, the side effect profile in clinical applications for human cancer therapy is important, as these bacteria are pathogens [5-7].

The health and nutritional benefits of orally administered probiotic lactic acid bacteria, such as *Lactobacillus *and *Bifidobacteria *species, which are a gram-positive and nonpathogenic, has begun to garner an increasing amount of attention [8,9].

Probiotics, such as lactic acid bacteria (LAB), are living microorganisms that affect the host in a beneficial manner by improving nutritional and microbial balance in the intestinal tract. These probiotic effects increase the immune response, reduce colon cancer, decrease serum cholesterol, and produce antimicrobial substances, such as bacteriocins that inhibit undesirable diarrhea- and disease-causing pathogens in the human intestine [10-18]. In addition, the dietary consumption of *B. lactis *HN019 enhances natural immunity in healthy elderly subjects [19-21]. Also, viable or heat-killed *Lactobacillus *and *Bifidobacterium *species, as well as certain of their cell components, are capable of stimulating the production of hydrogen peroxide, nitric oxide (NO), and cytokines, such as interleukin (IL)-6 and tumor necrosis factor (TNF)-α, in macrophage cell lines [22-24].

Further, several researchers have studied the anti-tumor effects exerted by lactic acid bacteria [25-38]. Sekine *et al*. detected anti-tumor activity in peptidoglycans isolated from the *B. infantis *strain, ATCC 15697, and Oda *et al*. reported anti-tumor activity in polysaccharide fractions originating from *Lactobacillus *cultures [30,32]. Glycoproteins detected in the supernatants of *Lactobacillus *cultures also have anti-tumor effects [33]. Many strains, including *L. rhamnosus GG, L. acidophilus*, *L. casei*, *B. longum*, *B. infantis*, *B. adolescentis*, and *B. breve*, suppress experimental colon tumor incidence [27,32-38], but the mechanisms of this tumor suppression are unclear [18,28,39].

Our goals were to evaluate the effects of *Bifidobacterium adolescentis *isolated from fecal samples of healthy young Koreans on immunostimulation and anti-proliferation of human colon cancer cell lines *in vitro*.

## Materials and methods

### Bacterial Culture

Fecal samples of 20 healthy Koreans (20–30 years old) were collected by BBL's anaerobic sample collection and transport system to maintain anaerobic conditions, and were used within 24 hr. Fecal samples were serially diluted 10-fold from 10^-1 ^to 10^-8^, and 100 μl was spread onto selective BL agar containing 5% sheep blood. After 48 hr of incubation in anaerobic conditions (Bactron Anaerobic Chamber, Sheldon Manufacturing Inc., USA) at 37°C, brown or reddish-brown colonies 2–3 mm in diameter were selected for further identification [40].

A fructose-6-phosphate phosphoketolase (F6PPK) test was performed [41] to ensure that the colonies selected were *Bifidobacteria*, and we analyzed the carbohydrate utilization pattern (Table 1). To identify the isolated *Bifidobacterium *spp. at the species level, 16S rRNA sequencing was performed by Bioleaders (Daejeon, Korea).

**Table 1:** Sugar utilization of *Bifidobacterium adolescentis *SPM

Sugar	*Bifidobacterium adolescentis*		
	
	SPM0212	SPM1005	SPM1601
L-Arabinose	-	+	-
D-Ribose	-	-	-
Xylose	+	+	+
Galactose	+	+	+
Fructose	+	+	+
Mannose	-	-	+
Mannitol	-	+	-
Sorbitol	-	-	-
Salicine	-	+	-
Cellobiose	-	-	+
Maltose	+	-	-
Lactose	+	+	-
Melibiose	+	+	+
Saccharose	+	+	+
Trehalose	+	+	-
Inuline	-	+	-
Melezitose	+	+	-
Raffinose	+	-	+
Starch	+	+	+
Gluconate	+	-	-

*B. adolescentis *SPM0212 was cultured at 37°C for 48 hr on general anaerobic medium (GAM, Nissui Pharm. Co. Ltd., Japan) under anaerobic conditions (90% N_2_, 5% H_2_, 5% CO_2_).

### Preparation of *B. adolescentis *SPM0212 Extract

For the preparation of *B. adolescentis *SPM0212 butanol extract, cultures were centrifuged (Vision, USA) at 13,000 rpm for 10 min, then the supernatant was removed and collected bacterial cell pellets were washed with autoclaved phosphate-buffered saline. These cell pellets were lyophilized, and this powder (0.095 g) was suspended in 50 ml of distilled water. Then, it was extracted with 50 ml of n-hexane or ethyl acetate or n-butanol. The BuOH fraction was visibly turbid. The organic solvent of extract was concentrated and removed using a rotary vacuum evaporation. The water, n-hexane, and EtOAc fraction was omitted because they showed low activity or no suppressive effect compared with BuOH fraction in the preliminary test.

### Cell Culture

The three human colon cancer cell lines (Table 2) and the murine macrophage cell line, RAW 264.7, was obtained from the Korean Cell Line Bank (Seoul, Korea) and the American Type Culture Collection (ATCC), respectively. Caco-2, HT-29, and SW480 cells were cultured in Roswell Park Memorial Institute-1640 (RPMI-1640) medium, including fetal bovine serum (FBS) and 1% (v/v) penicillin (10,000 U/ml)/streptomycin (10,000 U/ml) (P/S). RAW 264.7 cells were cultured in Dulbecco's modified Eagle's medium (DMEM) (with 10% FBS, 1% penicillin/streptomycin). All cultures were incubated at 37°C in a humidified atmosphere with 5% CO_2_. After they were grown to confluence in 75 cm^2 ^tissue culture flasks (NunC, Denmark), cells were detached and transferred to new cell culture dishes in a trypsin-versene mixture (Cambrex Bio Science, USA). Cell number and viability were assessed by the trypan blue dye-exclusion method [42].

**Table 2:** Characteristics of cell lines used in this study (KCLB, Korean Cell Line Bank)

Cell line	Cell type	Origin	Growth property	KCLB (ATCC) No.
Caco-2	Epithelioid	Colonic adenocarcinoma	Adherent	KCLB 30037
HT-29	Epithelioid	Colonic adenocarcinoma	Adherent	KCLB 3003
SW480	Epithelioid	Colonic adenocarcinoma	Adherent	KCLB 10228

### Tumor Cell Proliferation by XTT Assay

Cell proliferation was quantified via an XTT assay (sodium 3-[1-(phenylaminocarbonyl)-3,4-tetrazolium]-bis(4-methoxy-6-nitro)benzene sulfonic acid hydrate). Cells were seeded on 96-well microplates (NunC, Denmark) at 3 × 10^3 ^cells/well and incubated for 72 hr with the test compounds. Control was only cells (no treated). The butanol extract (no cells) was not tested. The samples were then incubated with 50 μl of XTT solution (1 mg/ml) for 6 hrs and measured with an ELISA reader (Molecular Devices, USA) at 490 nm.

### Tumor Necrosis Factor-α (TNF-α) Quantification

RAW 264.7 (1 × 10^5 ^cells/ml), LPS (*Escherichia coli *O127:B8 Westphal type, 100 ng/mL), and test samples (12.5, 25, 50, 100, 200 μg/ml) were prepared as treated groups and incubated for 48 hr. Following incubation, TNF-α secretion was assessed with an OPTEIA™ Mouse TNF-α kit (Pharmingen, San Diego, CA, USA) in accordance with manufacturer's protocol. Briefly, the sample and recombinant standards were added to antibody-coated plates and incubated for 2 hr. TNF-α was detected via the addition of horseradish peroxidase-conjugated, streptavidin-labeled antibodies. Color was developed using tetramethylbenzidine (TMB) (BD Biosciences, Pharmingen, USA) for 30 min and the absorbance was recorded at 450 nm.

### Nitric Oxide Assay

RAW 264.7 cells (1 × 10^6 ^cells/ml), LPS (50 ng/ml), and test samples (12.5, 25, 50, 100, 200 μg/ml) were prepared and incubated overnight. One hundred microliters from the surface of cultures was transferred into a new plate and the equivalent amount of Griess reagent was added (Stock-1: 0.2% naphylendia HCl, Stock-2: 2% sulfanilamide in 5% H_3_PO_4_). This plate was then incubated for 10 min at RT and measured by an ELISA reader at 540 nm. Standard calibration curves were prepared using sodium nitrite as a standard.

### Effect of *B. adolescentis *SPM0212 on Macrophage Morphology

RAW 264.7 cells (1 × 10^3 ^cells/well) were cultured in sterile glass-slide chambers for 48 hr. The culture medium was removed, and the cells were treated with either LPS (100 ng/ml) or samples of *B. adolescentis *SPM0212 (12.5, 25, 50, 100, 200 μg/ml) for 48 hr. Following treatment, the culture supernatant was removed, and the cells were fixed and stained in Diff Quick Solution (Baxter, Houston, TX). Macrophage morphology was observed using a light microscope (BX41, Olympus, Japan) at 400× magnification.

### Statistical Analysis

All data were expressed as the mean ± standard deviation (SD). For statistical evaluation of data, one-way ANOVA was applied using the program SPSS 13.0 for Windows. This was followed by post hoc comparisons using the Tukey's test. Significant differences were considered significant at *P *< 0.05.

## Results

### *B. adolescentis *Strains Inhibit the Growth of Colon Cancer Cell Lines

**Figure 1:**
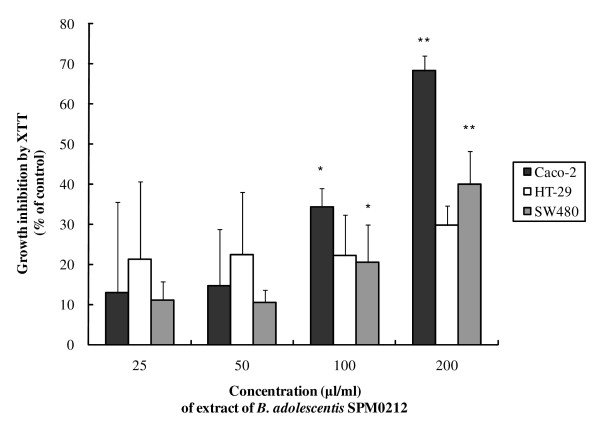
**Effects of growth inhibition by *B. adolescentis *SPM0212 on colon cancer cell lines (Caco-2, HT-29 and SW480)**. The cells (1 × 10^3 ^cells/well) were treated with *B. adolescentis *SPM0212 butanol extract (25, 50, 100, 200 μg/ml), and incubated for 72 hr at 37°C and 5.5% CO_2_. After adding 50 μl of the XTT labeling mixture, they were incubated for 6 hr at 37°C in 5.5% CO_2_. The absorbance was measured using an ELISA reader at 490 nm. The quantitative data were presented as means ± SD of three independent experiments. Control versus treatment groups, *p < 0.05; **p < 0.01.

To determine whether *B. adolescentis *strains inhibit the growth of the colon cancer cell lines, Caco-2, HT-29, and SW 480, cells were treated with 3 different *B. adolescentis *isolates, and XTT assays were performed. *B. adolescentis *SPM0212 exhibited the highest efficacy (data not shown). To further characterize the functional substances of *B. adolescentis *SPM0212, the cell lines were treated with the butanol extract of this strain. The butanol extract significantly inhibited proliferation of both Caco-2 and SW480 cell lines, with inhibition of Caco-2 and SW480 growth by 70% and 40%, respectively, at 200 μg/ml (Figure 1). Treatment with the same concentration of butanol extract also decreased proliferation of HT-29, but there was no significant difference.

### Effect of *B. adolescentis *SPM0212 on TNF-α and NO Production

**Figure 2:**
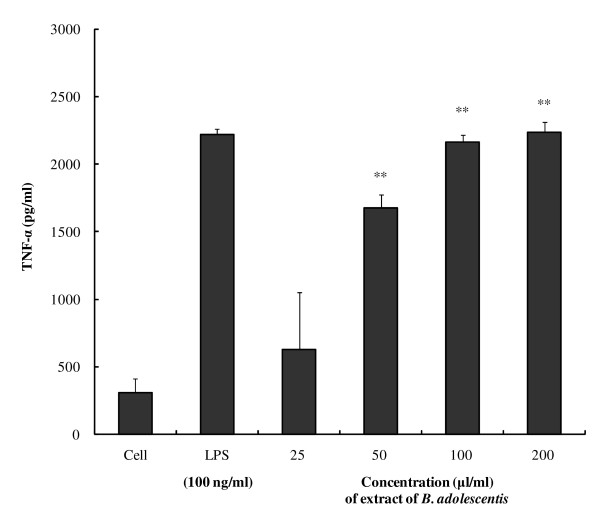
**Effects of *B. adolescentis *SPM0212 on TNF-α production from RAW 264.7 cells**. The cells (1 × 10^3 ^cells/well) were treated with LPS (100 ng/ml) or butanol extract of *B. adolescentis *SPM0212 (25, 50, 100, 200 μg/ml), and incubated for 48 hr at 37°C and 5.5% CO_2_. The extracellular levels of TNF-α in the culture media were determined by an ELISA reader at 450 nm. The quantitative data were presented as means ± SD of three independent experiments. Control versus *B. adolescentis *SPM0212 butanol extract, *p < 0.05; **p < 0.01.

**Figure 3:**
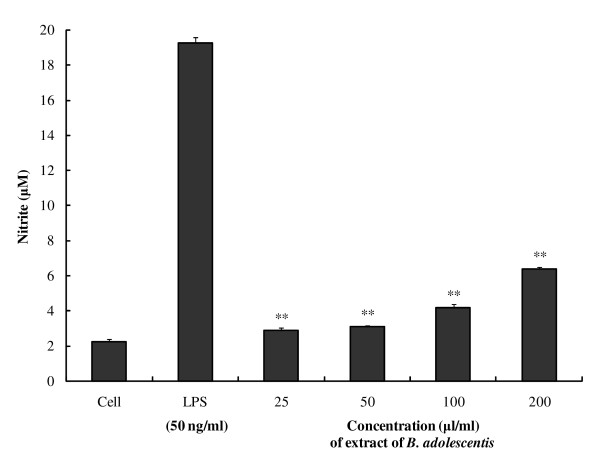
**Effects of *B. adolescentis *SPM0212 on NO production from RAW 264.7 cells**. The cells (1 × 10^3 ^cells/well) were treated with LPS (50 ng/ml) or butanol extract of *B. adolescentis *SPM0212 (25, 50, 100, 200 μg/ml), and incubated for 22 hr at 37°C and 5.5% CO_2_. Nitrite concentrations in the culture media were determined using Griess reagent assay and measured by ELISA reader at 540 nm. The quantitative data were presented as means ± SD of three independent experiments. Control versus *B. adolescentis *SPM0212 butanol extract, *p < 0.05; **p < 0.01.

Next, we examined the effects of *B. adolescentis *SPM0212 butanol extract on TNF-α and NO production by the macrophage RAW 264.7 cell line (Figure 2 and 3, respectively). *B. adolescentis *SPM0212 butanol extract significantly increased TNF-α production in a dose-dependent manner from 25 μg/ml to 200 μg/ml (Figure 2). Treatment with 200 μg/ml of butanol extract produced more TNF-α than LPS treatment, which was used as a positive control for macrophage activation. Treatment of RAW 264.7 cells with *B. adolescentis *SPM0212 butanol extract also increased production of NO (Figure 3). However, increases in TNF-α and NO production by *B. adolescentis *SPM0212 culture supernatant were not observed (data not shown).

### Morphology of RAW 264.7 cells treated with *B. adolescentis *SPM0212

**Figure 4:**
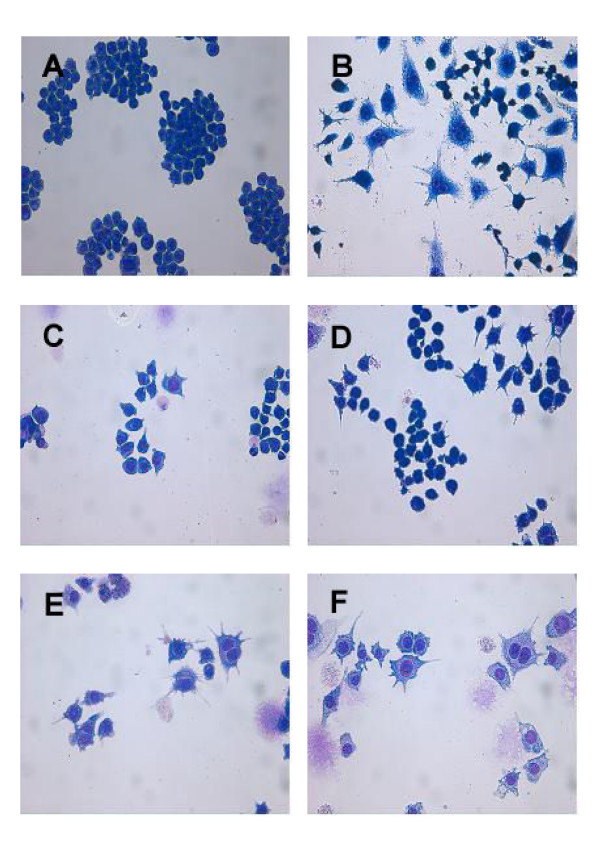
**Characterization of RAW 264.7 cells in response to butanol extract of *B. adolescentis *SPM0212**. RAW 264.7 cells (1 × 10^4 ^cells/well) were cultured on cover slips in the presence of different concentrations of butanol extract of *B. adolescentis *SPM0212 for 48 hr. The cells were fixed and stained in Diff-quick and observed under a light microscope at 400×. (A) Murine macrophage cells. (B) LPS (50 ng/ml). (C) Butanol extract of *B. adolescentis *SPM0212 (25 μg/ml). (D) Butanol extract of *B. adolescentis *SPM0212 (50 μg/ml). (E) Butanol extract of *B. adolescentis *SPM0212 (100 μg/ml). (F) Butanol extract of *B. adolescentis *SPM0212 (200 μg/ml).

Normal RAW 264.7 cells, when cultured in medium alone, look refractile and rounded morphology and do not spread over the surface (Figure 4A). Activated macrophages usually display a distinct morphology, which is similar to the dendritic cell. Exposure to LPS (50 ng/ml; the positive control) induced morphological alteration of the RAW 264.7 cells (Figure 4B). Treatment with *B. adolescentis *SPM0212 butanol extract caused RAW 264.7 cells to become larger and rougher in a dose-dependent manner, suggesting activation (Figure 4C–F).

## Discussion

*Bifidobacterium *spp., and LAB, are probiotic organisms in humans and stimulate immune function and anti-tumor effects [19-45]. Although the precise mechanisms by which LAB inhibit colon cancer are not known, several have been proposed: (a) enhancing the host immune response, (b) binding and degrading potential carcinogens, (c) alterations in the intestinal microflora that produce putative carcinogens, (d) production of anti-tumorigenic or anti-mutagenic compounds in the colon, and (e) alteration of metabolic activities of intestinal microflora [18,46-49].

LAB play an important role in the host immune system to produce anti-tumor effects [50-52]. Macrophages play a major role in the host defense against infection and tumor formation [53], and their function can be altered by a variety of stimulatory and suppressive signals and environmental factors. [54,55]. The production of nitric oxide (NO) and tumor necrosis factors (TNF-α) by macrophages mediate killing and growth inhibition of tumor cells, bacteria, fungi, and parasites [56]. TNF-α is a non-glycosylated 17 kDa protein that exists as a trimer in solution, has receptors on almost all somatic cells, regulates immune modulation, and is cytotoxic to tumor cells [57,58]. Also, TNF-α and reactive nitrogen intermediates play major roles in the *in vitro *anti-tumor activity of mouse peritoneal exudates from mice stimulated with wall peptidoglycan from *B. infantis *[32]. Therefore, cytokine production is a good measure of macrophage activation and further understanding of how *Bifidobacterium *affects the production of macrophage mediators may clarify how this strain affects immune function and tumor cells at the cellular level [22].

This study showed that the butanol extract of *B. adolescentis *SPM0212 increased secretion of TNF-α and NO from the macrophage RAW 264.7 cell line, as well as changed cell morphology. The butanol extract may contain key factors for increased macrophage activation and inhibition of tumor cell proliferation. Moreover, the butanol extract of *B. adolescentis *SPM0212 exerted direct anti-proliferative activity against three human colon cancer cell lines. We also observed that butanol extract of *B. adolescentis *SPM0212 – caused death of Caco-2, HT-29 and SW480 cells without any cytotoxicity to nonneoplastic epithelial cell (data not shown). Here, *B. adolescentis *SPM0212 potentiated TNF-production and may be beneficial in human intestinal tracts for immune reinforcement [59]. In contrast, most previously reported polysaccharides that exhibit anti-tumor activities did not directly inhibit the growth of tumor cells *in vitro*, but instead exerted anti-tumor activity by stimulating macrophages and immune responses. Therefore, the direct inhibitory effect by the butanol extract on tumor cell growth observed in this study is exceptional for polysaccharide biomaterials, but the active components remain to be elucidated. Further studies are needed to identify the effective components in the *B. adolescentis *SPM0212 butanol extract and will be required to clarify the precise mechanisms of this inhibition.

## Conclusion

*Bifidobacteria *strains have health-promoting effects. Our results showed that the butanol extract of *B. adolescentis *SPM0212, isolated from fecal samples of healthy young Koreans, markedly and dose-dependently decreased the proliferation of three human colon cancer cell lines, Caco-2, HT-29, and SW480. In addition, the butanol extract increased the production of the macrophage mediators, TNF-α and NO, and changed macrophage RAW 264.7 cell morphology. Therefore, this extract could potentially help to enhance the host immune system and improve human health by helping to prevent colon cancer as a biological response modifier (BRM).

## Competing interests

The authors declare that they have no competing interests.

## Authors' contributions

This study was conceived by NJH and designed by NJH and KJK. NJH and MJC were responsible for obtaining funding and sample collection. MJK and JHK carried out the extraction and separation. The cultures, XTT, TNF-α, and NO assay, analysis of morphology were done by DKL and SJ. DKL performed data analysis and wrote the draft of the manuscript. All authors read and approved the final manuscript.

## Pre-publication history

The pre-publication history for this paper can be accessed here:


